# *Things* seen *and unseen*: 1. Stunting and overweight/obesity are predominant malnutrition burdens of urban poor Nigerian adolescents

**DOI:** 10.5334/aogh.4550

**Published:** 2024-11-04

**Authors:** Chukwunonso ECC Ejike, Nneoma Uwadoka, Nkechi Igwe-Ogbonna

**Affiliations:** 1 Alex Ekwueme Federal University, Ndufu-Alike, Ebonyi State, Nigeria

**Keywords:** Adolescents, obesity, overweight, stunting, thinness

## Abstract

*Background:* Economic growth is associated with reductions in undernutrition. However, in developing countries, malnutrition still exists as a double burden. A better understanding of the dynamics of malnutrition in such societies as a means of aiding policymakers and implementers is thus needed.

*Objectives:* This study investigated the prevalence of malnutrition in Ebonyi State, Nigeria, and the role of socio‑economic status (SES) in driving it.

*Methods:* Standard protocols were used for all measurements. Overweight/obesity, stunting and thinness were defined using the simplified age‑ and gender‑specific height and body mass index (BMI) field tables of the World Health Organization (WHO).

*Results:* A total of 781 adolescents (65.4% female adolescents) from nine secondary schools were studied. Subjects in the rural and urban low SES groups were shorter than the others despite being older, and were shorter than the WHO reference cohort. In the general population, 3.2% (2.0% for girls and 5.6% for boys) were stunted. Urban low SES boys had the highest prevalence of stunting (18.6%). Thinness was found in 2.6% (7.4% for girls and 2.2% for boys) of the general population. It affected rural female adolescents (16.9%) more than the others and, as with stunting, was absent in the urban upper SES group. Overweight/obesity was found in 13.8% (12.5% for girls and 16.3% for boys) of the general population. It was highest amongst the urban upper SES group (35.9%) and absent amongst rural male adolescents. Stunting coexisting with thinness or with overweight/obesity was found in 0.8% and 0.25% of the general population, respectively.

*Conclusions:* Urban residence without improvements in SES is severely detrimental to the proper nutrition of adolescents.

## Introduction

Adolescence (age range: 10–19 years) is a critical phase in a child’s growth. It is a period of rapid physical, psychological, and cognitive development that can leave negative health signatures that track into adult life [1]. At adolescence, growth velocity is so high that up to 45% of potential skeletal growth, 50% of ideal adult weight and 15–25% of adult height are attained [2]. Adolescents are a large demographic, representing about 20% of the world’s population. It is estimated that 84% of adolescents live in developing countries, and a quarter of those live in sub‑Saharan Africa [3]. Despite the above, adolescents (relative to children) are underrepresented in health research, and this has consequences when it comes to understanding the dynamics of malnutrition in the age bracket, as well as the development of effective interventions [4, 5].

The rapid biological and psychosocial growth and development in adolescence comes with increased nutritional demands, which may lead to malnutrition [6], which presents as both under‑nutrition and over‑nutrition. Under‑nutrition refers to insufficient intake of dietary energy and nutrients to meet the body’s physiological needs. It manifests in children and adolescents as stunting (a chronic form, caused by a long period of inadequate nutrition leading to failure to attain optimum growth) or wasting/thinness (an acute form, caused by a recent food shortage and/or infectious diseases leading to rapid and significant weight loss) [7]. Over‑nutrition refers to excessive dietary energy intake relative to expenditure, which leads to excessive accumulation of fat and the attendant health impairments. It presents as overweight and obesity [8]. Malnutrition affects adolescents’ health through a variety of means. The short‑term consequences of under‑nutrition are poor academic performance, and frequent infections, while the long‑term consequences are poor general health and reduced economic productivity [9]. In contrast, over‑nutrition results in early development of non‑communicable diseases such as diabetes mellitus, hypertension, some cancers, etc. [10].

The World Health Organization (WHO) estimated in 2017 that more than 800 million children and adolescents globally are affected by stunting and wasting, and that sub‑Saharan Africa (SSA) bears a disproportionate burden of the disorders [10]. The prevalence of stunting in SSA in 2017 was 30.3% [11]. This occurred during a period when the number of overweight/obese children in the region rose steeply, from 5.4 million in 1990 to 10.3 million in 2015 [12]. In Nigeria, the prevalence of stunting and thinness in adolescents ranges from 3% to 33% and is affected by socio‑economic factors [1, 13–16]. A review of published papers between 1983 and 2013 reported that the prevalence of overweight and obesity in Nigerian adolescents was 1.0–8.6% and 0.0–2.8%, respectively [17]. The presence of both over‑ and under‑nutrition at the individual, household/family, or population level is described as the double burden of malnutrition (DBM) [18]. This is exacerbated by urbanisation and the increase in obesogenic environments that accompany it [15, 19], which often exist alongside food insecurity [20].

The ‘Conceptual Framework on the Determinants of Maternal and Child Nutrition’ from the United Nations Children’s Fund (UNICEF) described enabling determinants of malnutrition in children and adolescents to include political, financial, social, cultural, and environmental conditions. These enabling determinants influence children’s health through diets and care (immediate determinants) and family and community characteristics, such as maternal education (underlying determinants) [21]. Economic wellbeing is a clear determinant of nutritional status globally. Some studies have examined the association between economic growth and malnutrition in children. Harttgen *et al*. [22] and Vollmer *et al.* [23] after examining data from pooled Demographic and Health Surveys (DHS), each, found an inverse association between childhood stunting, underweight and wasting and economic growth. More recently, also using pooled DHS data, Yaya *et al.* [24], studied 20 African countries and reported a large inverse association between childhood stunting and economic growth. Buttner *et al*. [25], however, reported a weak association between economic growth and childhood malnutrition after an analysis of the DHS data of low‑ and middle‑income countries (LMICs) from 1990 to 2021.

Clearly, economic growth is associated with reductions in childhood stunting, wasting, and underweight, especially in LMICs [25]. Yet, in those countries, malnutrition still exists as a double burden, even within the same locality or household. This calls for a better understanding of the immediate and underlying determinants of malnutrition in societies as a means of guiding policymakers and programme implementers, if the ‘ending malnutrition in all of its forms by 2030’ goal of the Sustainable Development Goals is to be met. Previous studies had examined the subject by (predominantly) studying people living in different cities or states in Nigeria [16, 26]. The dynamics of the double burden of malnutrition within the same locality has scarcely been studied. In a study using school fees as a proxy for economic wellbeing, Ejike [15] showed that malnutrition affected the urban poor disproportionately, relative to the rural dwellers, even when they lived in close proximity. This study extends the investigation by examining the prevalence of under‑ and over‑nutrition in Abakaliki and Ikwo, and the role of socio‑economic status (SES) in driving it, by comparing data from urban dwellers disaggregated along SES lines to those of their rural‑dwelling counterparts. Hopefully, the findings will help policymakers and programme implementers in the drive to achieve the SDGs.

## Subjects and Methods

### Location and subjects

**Figure 1:**
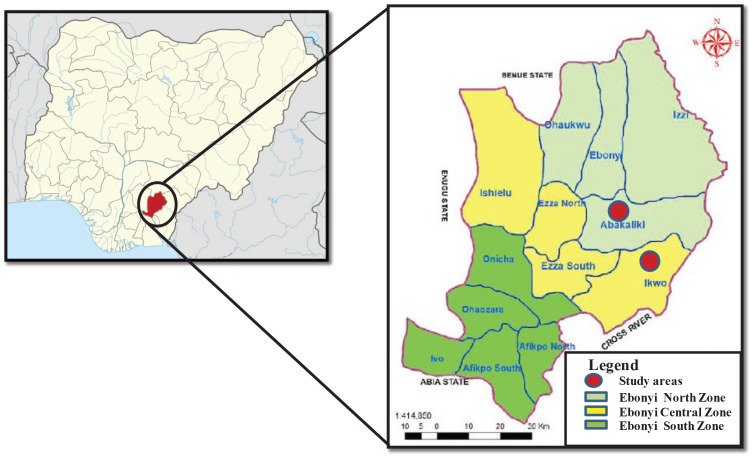
Map of Ebonyi State showing the study areas.

This was a cross‑sectional study of children and adolescents in Ebonyi State, Nigeria. Ebonyi is one of the five states in the South‑East geo‑political zone of Nigeria, predominantly populated by the Igbo ethnic group. It is the poorest state in the zone and the fourth poorest in Nigeria [27]. School‑going children and adolescents were recruited from selected secondary schools in Abakaliki and Ikwo. Abakaliki is the capital of Ebonyi State and an urban city. Ikwo is a rural agrarian local government area (LGA) in the state. Though Abakaliki and Ikwo are in two different geopolitical areas of the state, both LGAs are contiguous (Figure 1). Schools were purposively selected from the school’s list, and the principals of the schools were written formally, seeking permission to conduct the study in their schools. Fees charged by the schools were used as a proxy for SES in the urban area. Consequently, schools where tuition is free or less than ₦60,000.00 (∽$40) per annum were chosen to represent urban low SES, those that charge ₦90,000–150,000 (∽$60–100) per annum were chosen to represent urban middle SES and those that charge more than ₦300,000 (∽$200) per annum were chosen to represent the urban upper SES.

A total of nine schools (56.3% of those written to) – five schools in the rural area and four others in the urban area – whose principals gave written consent were chosen for the study. Letters were written to the parents/guardians of the students, informing them of the objectives of the study and methods to be used, and seeking their consent to allow their children/wards participate in the study. Only students whose parents/guardians gave written consent were allowed to participate in the study. Students who had any overt signs of ill‑health or physical deformities that could make anthropometric measurements difficult were excluded from the study. A total of 781 subjects (65.4% female adolescents), distributed as follows, were effectively recruited for the study: rural dwellers, 201 subjects (79.6% female adolescents); urban low SES, 182 subjects (67.6% female adolescents); urban middle SES, 306 subjects (58.2% female adolescents); and urban upper SES, 92 subjects (54.3% female adolescents).

### Methods

Self‑reported date of birth was recorded for each subject, and their ages determined. Height was measured (with the subject standing on bare feet) using a stadiometre, and recorded to the nearest 0.5 cm. Weight was measured (with the subject bare foot and wearing only their school uniform) using an electronic weighing balance, and recorded to the nearest 0.1 kg. Each morning, the weight scales were appropriately calibrated before use according to the manufacturer’s instructions. Waist circumference (WC) was measured to the nearest 0.5 cm using an inelastic measuring tape passed round the iliac crest and the naval, with the subject at the end of normal expiration. Hip circumference (HC) was measured to the nearest 0.5 cm using an inelastic measuring tape passed round the widest part of the buttocks. NWU and NSI‑O took all measurements in all locations.

From the height and weight data, body mass index (BMI) was calculated using the formula BMI = Weight (kg)/[Height (m)]^2^. Waist‑to‑height ratio (WHtR) was obtained by dividing the waist circumference of each subject by their height. Waist‑to‑hip ratio (WHpR) was obtained by dividing the waist circumference of each subject by their hip circumference. The study protocol was prepared in accordance with the Helsinki Declaration and was approved by the Board of the Department of Biochemistry, Alex Ekwueme Federal University, Ndufu‑Alike, Ebonyi State. Though stationery was given to participants as a token of appreciation, no honoraria were paid to them.

### Definitions

Overweight/obesity, stunting and thinness were defined using the simplified age‑ and gender‑specific height and BMI field tables of the World Health Organization (available at https://www.who.int/tools/growth-reference-data-for-5to19-years) [28]. Overweight/obesity and thinness were defined as BMI‑for‑age > 85th percentile and BMI‑for‑age < 5th percentile of the sex‑specific WHO reference tables, respectively. Stunting was defined as height‑for‑age < third percentile of the sex‑specific WHO reference tables.

### Data analysis

The data generated were subjected to basic descriptive statistical analysis and were reported as mean ± standard deviation for continuous data and percentages for categorical data. Differences between means for continuous data were separated by one‑way analysis of variance (ANOVA; with multiple comparisons where necessary). Significant differences between means were determined using a threshold of *P* < 0.05. The results are presented in tables or figures generated using Microsoft Office applications.

## Results

Rural girls and boys and their counterparts with urban low socio‑economic status had an average age of approximately 15 years, and there were no statistically significant differences (*P* > 0.05) between their ages when compared along sex lines. However, the rural dwellers were significantly (*P* < 0.05) older than their counterparts with urban middle‑ and urban‑upper socio‑economic status, who had average ages of approximately 13 years and 12 years, respectively (Table 1).

**Table 1:** Age and anthropometric indicators of nutritional status in the studied population.

	RURAL			URBAN LOW SES			URBAN MIDDLE SES			URBAN UPPER SES		
	FEMALE (160)	MALE (41)	*P*‑VALUE (FEMALE VERSUS MALE)	FEMALE (123)	MALE (59)	*P*‑VALUE (FEMALE VERSUS MALE)	FEMALE (178)	MALE (128)	*P*‑VALUE (FEMALE VERSUS MALE)	FEMALE (50)	MALE (42)	*P*‑VALUE (FEMALE VERSUS MALE)
Age	15.0 ± 1.9	15.2 ± 2.0	0.503	14.9 ± 1.8	15.8 ± 1.5	0.001	12.7 ± 1.5	12.9 ± 1.7	0.226	11.9 ± 2.0	11.7 ± 1.6	0.673
***P*‑value (rural versus others as per sex)**				0.390	0.224		<0.001	<0.001		<0.001	<0.001	
Weight	45.6 ± 8.2	48.0 ± 10.9	0.187	50.7 ± 8.2	54.7 ± 9.0	0.151	50.4 ± 11.0	48.5 ± 11.7	0.123	53.4 ± 16.9	48.0 ± 11.0	0.014
***P*‑value (rural versus others as per sex)**				<0.001	0.002		<0.001	0.793		<0.001	0.992	
Height	156.8 ± 7.1	161.1 ± 11.5	0.009	157.6 ± 8.2	161.7 ± 9.1	0.005	158.7 ± 8.8	160.7 ± 13.1	0.068	158.1 ± 11.0	155.7 ± 10.1	0.240
***P*‑value (rural versus others as per sex)**				0.505	0.742		0.073	0.809		0.407	0.010	
BMI	18.5 ± 2.6	18.3 ± 2.5	0.706	20.4 ± 3.1	20.8 ± 2.6	0.367	19.9 ± 3.5	18.6 ± 2.9	<0.001	21.1 ± 5.3	19.7 ± 3.5	0.039
***P*‑value (rural versus others as per sex)**				<0.001	<0.001		<0.001	0.573		<0.001	0.044	
WC	69.2 ± 5.4	70.5 ± 6.0	0.267	67.3 ± 5.4	68.5 ± 6.0	0.242	68.3 ± 7.0	67.4 ± 7.0	0.261	71.9 ± 10.2	70.2 ± 7.7	0.232
***P*‑value (rural versus others as per sex)**				0.013	0.133		0.195	0.009		0.013	0.862	
HC	82.8 ± 7.3	82.6 ± 7.5	0.956	92.2 ± 7.3	86.6 ± 7.5	0.227	88.0 ± 10.9	84.5 ± 9.8	0.306	95.2 ± 19.2	88.6 ± 10.2	0.291
***P*‑value (rural versus others as per sex)**				0.007	0.495		0.109	0.714		0.010	0.347	
WHpR	0.84 ± 0.05	0.86 ± 0.04	0.434	0.78 ± 0.08	0.79 ± 0.05	0.519	0.79 ± 0.05	0.80 ± 0.04	0.605	0.77 ± 0.07	0.80 ± 0.05	0.271
***P*‑value (rural versus others as per sex)**				<0.001	0.014		0.001	0.016		<0.001	0.032	
WHtR	0.44 ± 0.03	0.44 ± 0.03	0.561	0.43 ± 0.04	0.42 ± 0.03	0.501	0.43 ± 0.04	0.42 ± 0.04	0.023	0.45 ± 0.06	0.45 ± 0.05	0.775
***P*‑value (rural versus others as per sex)**				0.002	0.012		0.011	0.013		0.041	0.088	

Data on anthropometric measurements and their derivatives are shown in Table 1. Urban low SES male adolescents were the heaviest of the male adolescents, with an average weight of 54.7 ± 9.0 kg, which was significantly (*P* < 0.05) higher than the others. The average weights of the other groups compared with the rural boys were similar ( *P* > 0.05). The urban upper SES girls were the heaviest female adolescents, with an average weight of 53.4 ± 16.9 kg. All the urban subgroups had girls with an average weight that was significantly higher (*P* < 0.05) than that of the rural girls. Compared along sex lines, all the urban subgroups (except for urban middle SES boys) had significantly higher (*P* < 0.05) BMI compared with their rural counterparts. Compared with the rural girls, the mean waist circumference of the urban low, urban middle and urban upper SES girls were significantly lower (*P* < 0.05), similar (*P* > 0.05) and higher (*P* < 0.05), respectively. The values for WC for boys were similar (*P* > 0.05), except for the urban middle SES boys who had significantly lower (*P* < 0.05) mean WC values compared with the rural boys. Only urban low and urban upper SES girls had significantly higher (*P* < 0.05) mean hip circumference (HC) values compared with the rural girls. The others, including the boys, had statistically similar (*P* > 0.05) mean HC values. Mean waist‑to‑hip ratios (WHpR) were significantly higher (*P* < 0.05) in rural dwellers, irrespective of sex, compared with the other SES groups. Mean waist‑to‑height ratios (WHtR) of the girls (compared with the rural dwellers) were significantly (*P* < 0.05) lower in the urban low and middle SES groups, but higher in the urban upper SES group. For the boys, the WHtR were significantly lower (*P* < 0.05) in all the urban SES groups, except the upper SES group, where they were statistically similar (*P* > 0.05) to the mean values for the rural boys (Table 1).

**Figure 2:**
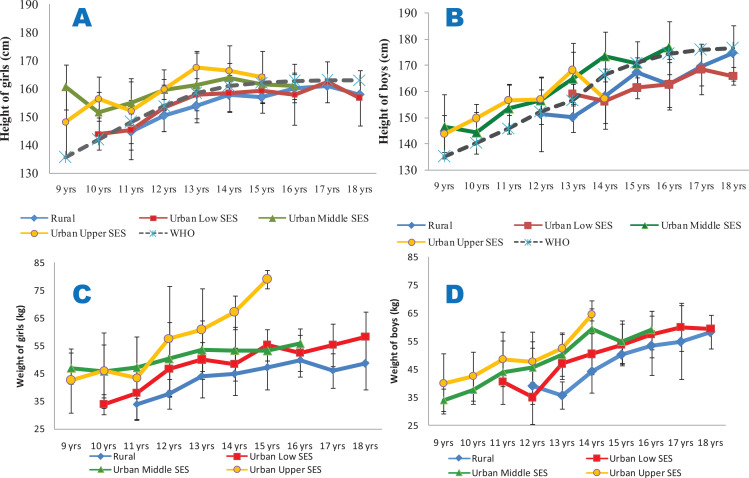
Mean heights (**2A** and **2B**) and weights (**2C** and **2D**) of the studied population. **2A** (height for girls) and **2B** (height for boys) are compared with the 85th percentiles of the WHO sex‑specific height reference dataset; **2C** (weight for girls) and **2D** (weight for boys). All the data are disaggregated as per place of residence and socio‑economic status.

**Figure 3:**
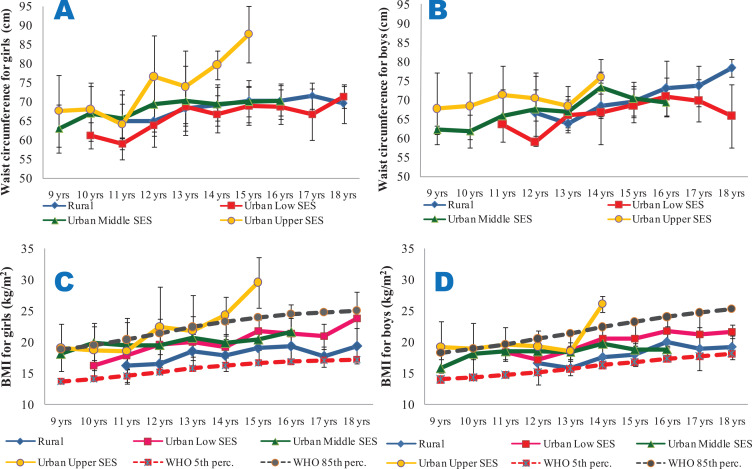
Mean waist circumference (**3A** and **3B**) and BMI (**3C** and **3D**) of the subjects. **3A** and **3B** are for waist circumferences for girls and boys, respectively. **3C** (BMI for girls) and **3D** (BMI for boys) are compared with the 5th and 85th percentiles of the WHO sex‑specific BMI reference dataset. All the data are disaggregated as per place of residence and socio‑economic status.

Rural and urban low SES girls and boys consistently were shorter than the 85th percentile of the WHO reference data for all the ages studied. The urban middle and upper SES girls and boys, however, were consistently taller than the reference age‑matched groups (Figure 2A and B). Urban upper SES girls and boys had higher weights (Figure 2C and D) and waist circumferences (Figure 3A and B) compared with the other groups. Conversely, the rural dwellers had lower mean weights than the others, followed by the urban low SES group. Compared with the others, the urban low SES group had the lowest mean WC values, followed by the rural dwellers. Clearly, rural and urban poor SES groups had lower growth performance indicators, irrespective of age, compared with their counterparts of urban middle and upper SES (Figures 2 and 3).

**Figure 4:**
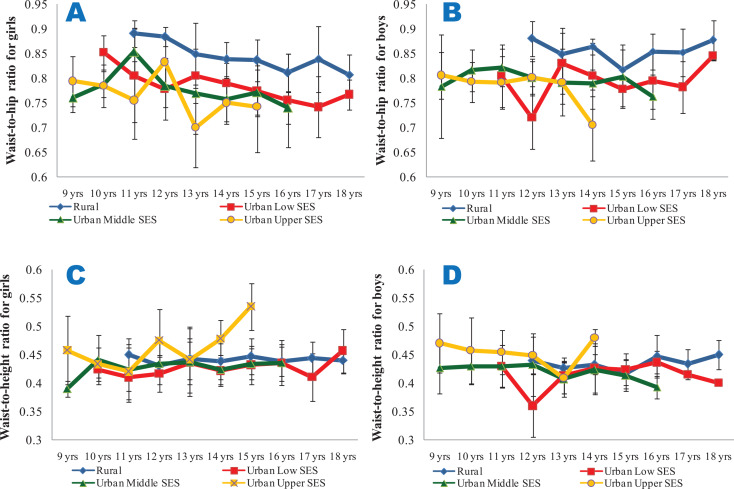
Mean waist‑to‑hip ratios (**4A** and **4B**) and waist‑to‑height ratios (**4C** and **4D**) of the subjects. **4A** and **4C** show data for girls, while **4B** and **4D** show data for boys. All the data are disaggregated as per place of residence and socio‑economic status.

From Figure 3C and D, it is seen that the BMI of the girls and boys were largely within the cut‑off points for overweight and thinness prescribed by the WHO for all the ages and SES groups, with the exception of the urban upper SES groups, particularly for ages 14 and 15 years. Though the mean WHpR values were generally highest in the rural girls and boys (irrespective of age) and lowest in their urban upper SES counterparts, the values were all lower than 1. However, the mean WHtR values were highest in the urban upper SES groups, and exceeded 0.5 only for the urban upper SES girls at the age of 15 years (Figure 4).

In the general population, 3.2% (2.0% for girls and 5.6% for boys) were stunted (Table 2). When disaggregated on the basis of social stratification, stunting was found in 4.0% of rural dwellers (3.1% for girls and 7.3% for boys), 8.8% of the urban low SES group (4.1% for girls and 18.6% for boys), 0.3% of the urban middle SES group (0.0% for girls and 0.3% for boys) and 0.0% of the urban upper SES group. The prevalence of stunting was highest amongst urban low SES subjects, reaching 33.3% amongst 18‑year‑old female adolescents and 37.5% amongst 16‑year‑old male adolescents of the SES group (Table 3).

**Table 2:** Prevalence of malnutrition in the general population (irrespective of SES).

AGE (YEARS)	POPULATION DISTRIBUTION			PREVALENCE OF THINNESS			PREVALENCE OF STUNTING			PREVALENCE OF OBESITY/OVERWEIGHT		
	FEMALE (*N*)	MALE (*N*)	ALL (*N*)	FEMALE (%)	MALE (%)	ALL (%)	FEMALE (%)	MALE (%)	ALL (%)	FEMALE (%)	MALE (%)	ALL (%)
**9**	10	7	17	0.0	0.0	0.0	0.0	0.0	0.0	30.0	42.9	35.3
**10**	32	18	50	0.0	0.0	0.0	0.0	0.0	0.0	31.3	27.8	30.0
**11**	49	40	89	4.1	5.0	2.2	0.0	0.0	0.0	24.5	27.5	25.8
**12**	69	36	105	7.2	22.2	7.6	1.4	0.0	1.0	14.5	19.4	16.2
**13**	98	45	143	7.1	15.6	4.9	4.1	2.2	3.5	13.3	11.1	12.6
**14**	88	36	124	11.4	30.6	8.9	2.3	8.3	4.0	6.8	16.7	9.7
**15**	58	31	89	5.2	12.9	4.5	0.0	6.5	2.2	10.3	9.7	10.1
**16**	66	33	99	6.1	15.2	5.1	3.0	21.2	9.1	4.5	6.1	5.1
**17**	25	19	44	16.0	36.8	15.9	0.0	10.5	4.5	0.0	10.5	4.5
**18**	10	4	14	30.0	75.0	21.4	10.0	0.0	7.1	10.0	0.0	7.1
**19**	6	1	7	0.0	0.0	0.0	0.0	0.0	0.0	0.0	0.0	0.0
**Total**	511	270	781	2.2	7.4	2.6	2.0	5.6	3.2	12.5	16.3	13.8

**Table 3:** Prevalence of stunting (low height for age) in the studied population, disaggregated by SES.

AGE (YEARS)	RURAL (*N* = 201)				URBAN LOW (*N* = 182)				URBAN MIDDLE (*N* = 306)				URBAN UPPER (*N* = 92)			
	FEMALE	%	MALE	%	FEMALE	%	MALE	%	FEMALE	%	MALE	%	FEMALE	%	MALE	%
**9**	–	–	–	–	–	–	–	–	–	–	–	–	10	0.0	7	0.0
**10**	–	–	–	–	–	–	–	–	18	0.0	10	0.0	14	0.0	8	0.0
**11**	–	–	–	–	–	–	–	–	45	0.0	33	0.0	4	0.0	7	0.0
**12**	13	0.0	3	0.0	12	8.3	1	0.0	40	0.0	23	0.0	4	0.0	9	0.0
**13**	27	11.1	5	0.0	25	4.0	3	0.0	37	0.0	29	3.4	9	0.0	8	0.0
**14**	35	2.9	8	12.5	23	4.3	9	22.2	25	0.0	17	0.0	5	0.0	2	0.0
**15**	26	0.0	6	0.0	20	0.0	15	13.3	8	0.0	9	0.0	4	0.0	1	0.0
**16**	36	2.8	10	10.0	25	4.0	16	37.5	5	0.0	7	0.0	–	–	–	–
**17**	10	0.0	6	16.7	15	0.0	13	7.7	–	–	–	–	–	–	–	–
**18**	7	0.0	2	0.0	3	33.3	2	0.0	–	–	–	–	–	–	–	–
**19**	6	0.0	1	0.0	–	–	–	–	–	–	–	–	–	–	–	–
**Total**	160	3.1	41	7.3	123	4.1	59	18.6	178	0.0	128	0.8	50	0.0	42	0.0
**All (female + male)**	201 (4.0%)				182 (8.8%)				306 (0.3%)				92 (0.0%)			

N/B: Percentages are calculated as per place of residence.

Thinness was found in 2.6% (7.4% for girls and 2.2% for boys) of the general population (Table 2). When disaggregated, thinness was found in 14.9% (16.9% for girls and 7.3% for boys) of rural dwellers, 4.4% (4.1% for girls and 5.1% for boys) of the urban low SES group, 2.9% (3.4% for girls and 2.3% for boys) of the urban middle SES group and 0.0% of the urban upper SES group. Again, thinness was highest amongst urban low SES subjects, reaching 33.3% amongst 18‑year‑old female adolescents, and the rural dwellers, where it reached 15.4% amongst 17‑year‑old male adolescents (Table 4).

**Table 4:** Prevalence of thinness (low BMI for age) in the studied population, disaggregated by SES.

AGE (YEARS)	RURAL (*N* = 201)				URBAN LOW (*N* = 182)				URBAN MIDDLE (*N* = 306)				URBAN UPPER (*N* = 92)			
	FEMALE	%	MALE	%	FEMALE	%	MALE	%	FEMALE	%	MALE	%	FEMALE	%	MALE	%
**9**	–	–	–	–	–	–	–	–	–	–	–	–	10	0.0	7	0.0
**10**	–	–	–	–	–	–	–	–	18	0.0	10	0.0	14	0.0	8	0.0
**11**	–	–	–	–	–	–	–	–	45	4.4	33	0.0	4	0.0	7	0.0
**12**	13	23.1	3	0.0	12	8.3	1	0.0	40	2.5	23	13.0	4	0.0	9	0.0
**13**	27	18.5	5	0.0	25	4.0	3	0.0	37	2.7	29	0.0	9	0.0	8	0.0
**14**	35	20.0	8	12.5	23	4.3	9	0.0	25	8.0	17	0.0	5	0.0	2	0.0
**15**	26	11.5	6	0.0	20	0.0	15	6.7	8	0.0	9	0.0	4	0.0	1	0.0
**16**	36	8.3	10	10.0	25	4.0	16	0.0	5	0.0	7	0.0	–	–	–	–
**17**	10	40.0	6	16.7	15	0.0	13	15.4	–	–	–	–	–	–	–	–
**18**	7	28.6	2	0.0	3	33.3	2	0.0	–	–	–	–	–	–	–	–
**19**	6	0.0	1	0.0	–	–	–	–	–	–	–	–	–	–	–	–
**Total**	160	16.9	41	7.3	123	4.1	59	5.1	178	3.4	128	2.3	50	0.0	42	0.0
**All (female + male)**	201 (14.9%)				182 (4.4%)				306 (2.9%)				92 (0.0%)			

N/B: Percentages are calculated as per place of residence.

From Table 2, it is seen that overweight/obesity was found in 13.8% (12.5% for girls and 16.3% for boys) of the general population. Disaggregated, overweight/obesity was found in 2.5% (3.1% for girls and 0.0% for boys) of rural dwellers, 8.2% (6.5% for girls and 11.9% for boys) of the urban low SES group, 18.0% (19.1% for girls and 16.4% for boys) of the urban middle SES group and 35.9% (34.0% for girls and 38.1% for boys) of the urban upper SES group (Table 5). In all the age groups of the urban upper SES group (except for 13‑year‑old male adolescents), overweight/obesity exceeded 20%. Indeed, in the same SES group, the prevalence of overweight/obesity reached or exceeded 60% for the ages of 15 and 16 years, irrespective of sex. Conversely, overweight/obesity was not found amongst rural boys.

**Table 5:** Prevalence of overweight/obesity (high BMI for age) in the studied population, disaggregated by SES.

AGE (YEARS)	RURAL (*N* = 201)				URBAN LOW (*N* = 182)				URBAN MIDDLE (*N* = 306)				URBAN UPPER (*N* = 92)			
	FEMALE	%	MALE	%	FEMALE	%	MALE	%	FEMALE	%	MALE	%	FEMALE	%	MALE	%
**9**	–	–	–	–	–	–	–	–	–	–	–	–	10	30.0	7	42.9
**10**	–	–	–	–	–	–	–	–	18	38.9	10	20.0	14	21.4	8	37.5
**11**	–	–	–	–	–	–	–	–	45	24.4	33	24.2	4	25.0	7	42.9
**12**	13	0.0	3	0.0	12	25.0	1	0.0	40	15.0	23	13.0	4	25.0	9	44.4
**13**	27	7.4	5	0.0	25	8.0	3	0.0	37	18.9	29	17.2	9	22.2	8	0.0
**14**	35	2.9	8	0.0	23	0.0	9	11.1	25	8.0	17	17.6	5	60.0	2	100.0
**15**	26	3.8	6	0.0	20	5.0	15	13.3	8	0.0	9	0.0	4	100.0	1	100.0
**16**	36	2.8	10	0.0	25	4.0	16	12.5	5	20.0	7	0.0	–	–	–	–
**17**	10	0.0	6	0.0	15	0.0	13	15.4	–	–	–	–	–	–	–	–
**18**	7	0.0	2	0.0	3	33.3	2	0.0	–	–	–	–	–	–	–	–
**19**	6	0.0	1	0.0	–	–	–	–	–	–	–	–	–	–	–	–
**Total**	160	3.1	41	0.0	123	6.5	59	11.9	178	19.1	128	16.4	50	34.0	42	38.1
**All (female + male)**	201 (2.5%)				182 (8.2%)				306 (18.0%)				92 (35.9%)			

N/B: Percentages are calculated as per place of residence.

Stunting and thinness were found to coexist in 0.8% (1.0% for girls and 0.4% for boys) in the general population as well as in 2.5% of rural female adolescents, 2.4% of rural male adolescents and 0.8% of urban low SES female adolescents, but not the other groups. All the subjects who had both stunting and thinness were aged 13–15 years. Stunting coexisting with overweight/obesity was found only in the urban low SES group, where it affected 0.8% and 1.7% of female and male adolescents, respectively, all aged 17–18 years. The prevalence of stunting coexisting with overweight/obesity in the general population was therefore 0.3% (0.02% for girls and 0.4% for boys).

## Discussion

The finding that urban middle and upper SES groups were younger than rural and urban low SES groups was expected. Middle and upper SES parents in Nigeria often enrol their children early in primary schools, and then rush them through school, often skipping classes. The result is that the children of urban middle and upper SES parents often get to secondary school before the age of 12 years and graduate before they are 18 years old. Rural and urban poor parents are more likely to stick to the expected ages for enrolment of children in Nigerian primary and secondary schools (6 years and 12 years, respectively) often because their children attend public schools where such national guidelines are more likely to be enforced. This made it difficult to find and enrol subjects younger than 12 years in the rural and urban lower SES groups or those older than 16 years in the urban upper SES groups for this study.

Subjects in the rural and urban low SES groups (on the average) were consistently shorter than the other age groups when the means were compared as a whole or when disaggregated on the basis of their ages, despite their being older. Furthermore, they were shorter than the WHO reference dataset, at the studied ages, clearly failing to reach the expected vertical growth milestones. This growth faltering affected the girls more than the boys and would have contributed to the high prevalence rates of stunting found in this study. Indeed, the boys were significantly taller than the girls only in the rural and urban low SES groups. In the urban middle and upper SES groups where the subjects surpassed the heights of the reference dataset, the mean heights of the boys and girls were statistically similar. This may be explained by the fact that girls experience the growth spurt earlier than boys, but boys often become taller in late adolescence [15, 29]. This fact appears masked in our data owing to our inability to enrol older adolescents in the urban upper SES group, as discussed earlier. The finding that rural and urban lower SES adolescents in Nigeria did not attain optimal growth may be attributed to nutritional deficiencies in childhood and adolescence and has been reported by previous Nigerian authors [1, 13, 26]. The rural and urban lower SES groups clearly had lower growth performance indicators, irrespective of age, compared with their counterparts of urban middle and upper SES.

In the general population studied, 3.2% (2.0% for girls and 5.6% for boys) were stunted. We found that urban low SES boys (followed by rural boys) had the highest prevalence of stunting. As much as 7.3% of rural male adolescents and 18.6% of urban low SES male adolescents, but none of urban upper SES groups, were stunted. The prevalence of stunting was greater in the mid‑ to late adolescence age bracket, reaching 37.5% amongst 16‑year‑old male adolescents of the urban low SES group. We had reported a prevalence of stunting in adolescents in Ajaokuta, Northern Nigeria, to be 16.2% (23.8% for boys and 4.8% for girls) [13]. Wariri *et al*. [16] reported stunting in Gombe (Northern Nigeria) and Uyo (Southern Nigeria) to affect 12.5% and 8.1% of adolescents, respectively. In Nsukka (Southern Nigeria), the prevalence of stunting amongst adolescents was 33.3% [14]. Gezaw *et al*. [3] reported that the pooled prevalence of stunting in Ethiopia was 20.1%, while amongst Indian adolescents, Pandurangi *et al*. [30] reported a prevalence of 27.4% for stunting. The prevalence of stunting in the general population studied appears quite smaller than values reported by other studies. However, when disaggregated, the prevalence of stunting in rural and urban low SES subjects, particularly the male adolescents, was within the ranges reported for Gombe and Uyo [16], but lower than the figures from Ethiopia [3] and India [30]. The absence of stunting in the urban upper SES group is indicative of the role of economic empowerment in preventing stunting. Furthermore, the presence of stunting in the urban lower SES group suggests that a family’s financial status, not mere urban residence, is a factor for consideration in designing nutrition interventions.

Thinness was found in 2.6% (7.4% for girls and 2.2% for boys) of the general population. Thinness affected rural female adolescents more (16.9%) than the others, and was absent in the urban upper SES group. Earlier, we had reported a child/adolescent thinness prevalence of 24.2% in boys and 19.2% in girls living in Umuahia [13] and 3.8% in adolescents (4.8% in boys and 2.4% in girls) living in Ajaokuta [1]. The prevalence of thinness in Nsukka was 31.0%, and none of the subjects were either overweight or obese [14]. Wariri *et al*. [16] found an overall prevalence of thinness of 11.98% and 5.3% in adolescents in Gombe and Uyo, respectively. Gezaw *et al*. [3] reported that the pooled prevalence of thinness in Ethiopia was 21.7%. Amongst Indian adolescents, Pandurangi *et al*. [30] reported a prevalence of 24.4% for thinness and noted that stunting affected more girls than boys and was more prevalent in late adolescence, whereas thinness was more prevalent in boys and in early adolescence. This agrees with our findings when it comes to our finding more cases of stunting in late adolescence; otherwise, the sex‑related distribution of the prevalence of stunting and thinness is at variance with our findings. Methodological and cultural differences may explain the noted divergence. The prevalence of stunting in this study is lower than all the referenced studies but is close to the 3.8% prevalence reported by Ejike *et al.* [1] in Ajaokuta and the 5.3% reported by Wariri *et al*. [16] for only Uyo. The rural female adolescents who had the highest prevalence of thinness in this study still had lower prevalence compared with most of the other studies from Nigeria and elsewhere. Given that thinness often reflects short‑term food insecurity, it is possible that adolescents in Ebonyi State, a largely agrarian state, had more reliable access to food (at least quantity‑wise). The period in which this study was conducted, September to November, coincides with the harvest season in Nigeria, when food is often abundant, and is similar to the period during which the study in Ajaokuta was conducted. This seasonal variation in food availability in rural and semi‑urban areas may explain the low prevalence of thinness reported in this study, and some of the observed variations in thinness between this and some earlier studies.

Our finding that stunting affected male adolescents more than female adolescents is in consonance with our previous reports [1, 13] and those of some authors [31–33] but not others [34]. Indeed, Wrottesley *et al*. [35] in a review of the nutritional status of adolescents from low‑ and middle‑income countries reported that boys more often than girls were stunted in West and Central African countries. Cultural practices that reserve more physically exerting tasks for male adolescents, and preferentially provide more food and snacks to girls (especially when the family is food insecure) may explain part of this observation. Additionally, there still persists, in many economically less developed societies, cultural practices which remain in the subconscious of even the educated and promote the idea that a plump woman will have better chances in the marriage market. Thus, mothers may deliberately give more food to the girls as snacks or in the kitchen while the food is being prepared, and this, too, can confer a growth advantage on them early in life. There are reports also that stunting and thinness were more prevalent in rural areas compared with urban areas [31, 35]. This would appear to be the case with our data without disaggregation of the urban dwellers. Disaggregation of the urban dwellers enabled us to see that the urban poor were indeed worse off than the rural dwellers with respect to stunting. This draws attention to the fact that place of residence may mask the deep nutritional inequality that exists amongst people of different SES groups who live in the same area. Thinness affected late adolescents more, reaching a prevalence of 40% and 33% amongst rural 17‑year‑old female adolescents and urban low SES 18‑year‑old female adolescents, respectively. This finding of stunting and thinness in mid‑ to late adolescence is in concordance with a previous report from two cities in Nigeria [16]. The aforementioned study reported that significantly more mid‑ to late adolescents were stunted and thin in both Gombe and Uyo. In Ethiopia, thinness was two‑fold more prevalent in early adolescents than in late adolescents, and this was attributed to faster growth and development in the phase of growth in the early ages of adolescence [3].

Overweight/obesity was found in 13.8% (12.5% for girls and 16.3% for boys) of the general population. Overweight/obesity was lowest amongst rural dwellers [2.5% (3.1% for girls and 0.0% for boys)] and highest amongst the urban upper SES group [35.9% (34.0% for girls and 38.1% for boys)]. In all the age groups of the urban upper SES group (except for 13‑year‑old male adolescents), overweight/obesity prevalence exceeded 20%. For Gombe and Uyo, Nigeria, a prevalence of overweight/obesity of 16.08% and 11.31%, respectively, were reported [16]. The prevalence of overweight/obesity in Ethiopia from a pooled analysis was 10.63% [3]. Amongst Indian adolescents, Pandurangi *et al*. [30] reported a prevalence of 5.9% for overweight/obesity. The prevalence values reported for the general population are higher than the value reported in India, but within the values reported by the cited recent studies in Nigeria and sub‑Saharan Africa, and are slightly higher than the values reported following a review of the literature on the subject in Nigeria between 1983 and 2013 [17]. This suggests a plausible increase in obesity prevalence in the last 10 years. The very high prevalence rates of overweight/obesity found in the urban upper SES group and its near absence in the rural group point to the role of economic wellbeing as a driver of obesity. Additionally, the double‑digit prevalence of overweight/obesity in the urban lower SES group suggests that lifestyle factors dominant in urban areas, and not just economic wellbeing, contribute significantly to overweight/obesity. The vulnerability of the urban poor to overweight/obesity despite the existence of stunting and thinness in the sub‑population may be due to early foetal and childhood nutritional deficiencies, which they may be compensating for as they experience a lifestyle transition that comes with urban living.

Our finding of a marginally higher prevalence of overweight/obesity amongst male adolescents agrees with a report from India [30], but with not others such as Rachmi *et al.* [31] or Hamann *et al.* [36], who reported that overweight/obesity was more prevalent in female adolescents. Indeed, Wrottesley *et al*. [35] in their review found that, in West and Central Africa, more female than male subjects were reported to be overweight/obese. Female preponderance of overweight/obesity in adolescents is fairly well established in the literature and the marginal difference observed in our study may not be sufficient to alter that position. It is often thought that boys engage in more physically exerting chores and play routines than girls and, as such, do not accumulate as much excess fats as girls. It appears, however, that this may be true in rural area and urban middle SES class homes, but not amongst the urban higher SES class families (where energy‑sparing devices are available and domestic workers and not children do the heavy lifting). Pandurangi *et al*. [30] additionally reported a higher prevalence of overweight/obesity in early adolescence. We found high obesity rates in the urban upper SES group but, incidentally, did not have sufficient subjects in the late adolescent ages of that group to support any meaningful comparisons. Our data agree in part with the reports of Rachmi *et al.* [31], who after studying data from countries in the Association of Southeat Asian Nations (ASEAN), found a higher prevalence of overweight/obesity in urban areas compared with rural areas.

Stunting and thinness were found to coexist in 0.8% (1.0% for girls and 0.4% for boys) in the general population, and in 2.5% of rural female adolescents, 2.4% of rural male adolescents and 0.8% of urban low SES female adolescents, but not the other groups. All the subjects who had both stunting and thinness were in the mid‑adolescence phase (13–15 years). Stunting coexisting with overweight/obesity was found only in the urban low SES group, where it affected 0.8% and 1.7% of female and male adolescents, respectively. The affected adolescents were all in the late adolescence phase (17–18 years). This describes a case of double burden of malnutrition that disproportionally affects the urban lower SES group at the individual level and the urban lower SES and rural groups at the community level. It is worthy of note, however, that the prevalence of the double burden of malnutrition (DBM) reported here is quite low. Wariri *et al*. [16] reported a prevalence of 8.0% for stunting coexisting with generalised obesity in Nigeria. It is reported that the DBM is 5.0% in Vietnam and up to 29.8% in Indonesia [31]. The urban poor appear trapped in the whirlwind of a rapidly transforming economy. The consequent nutritional epidemiological shift brings along with it an increased consumption of highly processed energy‑dense foods with low nutritional value, which the poor wrongly see as “rich people’s food” and therefore crave, despite their often chronic nutritional deficits (typically stunting). This is worsened by poor nutritional knowledge and the unguided exposure to mass media and the effects they have on cultural norms related to nutrition [19, 37]. This affects adolescents more, as they are known to be very susceptible to the adoption of unhealthy lifestyles when they come in contact with practices they perceive as “trending” [38, 39]. Sadly, the consequences of such adopted unhealthy lifestyles often persist into adulthood.

It appears obvious that the economic disposition of families is directly related to the nutritional status of their adolescents. Wrottesley *et al*. [35] noted that, in addition to understanding the determinants of malnutrition in populations, understanding differences between rural and urban dwellers is critical for developing and targeting interventions. Gezaw *et al*. [3] indicate that urban residence, in addition to having a family size of greater than five people, predicted stunting. We contend, however, that it may be wrong to construct poverty and its effect on child and adolescent nutrition as a rural versus urban matter. It is known that economic growth does not automatically reduce malnutrition in populations, irrespective of place of domicile. This is because income is rarely fairly distributed, and poor households might not necessarily prioritise investments in their children’s nutrition due to lack of knowledge [24, 25]. From our data, the urban poor had a higher burden of stunting and overweight/obesity (determined as single entities or as a double burden) compared with the rural dwellers. The rural dwellers, however, had more thin adolescents. In any case, thinness unlike stunting is not a chronic form of malnutrition and may reflect seasonal variations in access to food or in the rise of infectious agents.

The major message of this study is that the urban poor shares in the health challenges of overnutrition of the urban rich and undernutrition of the rural dwellers, resulting in their being worse off when nutrition is taken holistically. Migration to urban areas without the requisite economic wherewithal, as seen in the urban lower SES, is severely detrimental to the nutritional status of adolescents. Hamann *et al*. [36] reached a similar conclusion after studying adolescents in Nepal. Public health interventions targeting adolescents must abandon an omnibus system of nutrition messaging and deliberately target different SES groups with tailor‑made specific messages for each group, as their nutritional challenges clearly differ, and one message may not fit all. In line with the WHO’s position [18], double‑duty interventions, that is nutrition programmes and policies which are designed to simultaneously act on both under‑ and over‑nutrition, should be developed for specific SES groups in Ebonyi State in particular, and in Nigeria in general. Such programmes and policies will also likely find application in other low‑ and middle‑income countries.

### Limitations

This study is limited by the fact that only school‑going adolescents were enroled and studied. It would have benefitted from the enrolment of all eligible and willing adolescents in the study area. Logistical and funding constraints, however, did not permit this. Secondly, school fees were used as proxy for socio‑economic status, instead of direct determination of the socio‑economic status of the families. Apparently, some middle‑class parents may send their children to schools where no fees are paid or to those that charge very high fees, depending on the premium they place on the education of their children and their assessment of what quality education truly means. This may have led to a wrong characterisation of the SES of some subjects. However, we feel that the errors that could have resulted from this would be marginal and may not affect the overall conclusions from the data. Thirdly, the study would have benefitted from an in‑depth assessment of the food intake of the adolescents, even if from 1‑week recall assessments. It was, however, difficult to distract the students from their studies, and the principals would not allow us to spend a lot more time with them. Again, we opine that the manifest effects of nutrition as seen from nutritional status assessments are sufficient to reach reasonable conclusions for a cross‑sectional study such as this. Given our environment, we believe that the fairly large sample size, even after disaggregation into SES groups; our easy‑to‑implement measure of SES; and our robust findings and conclusions are important strengths of this study.

## Conclusion

This study investigated the prevalence of under‑ and over‑nutrition in Abakaliki and Ikwo, and the role of socio‑economic status in driving it. A total of 781 subjects (65.4% female adolescents) were effectively recruited and studied. Subjects in the rural and urban low SES groups (on the average) were consistently shorter than the others despite their being older, and were shorter than the WHO reference dataset at the different ages. Stunting affected male adolescents (particularly urban low SES boys) more than female adolescents, but thinness affected more female adolescents (particularly rural female adolescents) than male adolescents. Overweight/obesity was highest amongst the urban upper SES group. Stunting and thinness were absent in the urban upper SES group, while overweight/obesity was absent in rural boys. Stunting coexisting with thinness, on the one hand, and stunting coexisting with overweight/obesity, on the other hand, were found in a very small fraction of the rural and urban low SES groups. Clearly, the urban poor adolescent shares in the malnutrition woes of both the urban rich and the rural dwellers. Urban residence without improvements in SES is severely detrimental to the proper nutrition of adolescents.
